# PONDEROSA-C/S: client–server based software package for automated protein 3D structure determination

**DOI:** 10.1007/s10858-014-9855-x

**Published:** 2014-09-05

**Authors:** Woonghee Lee, Jaime L. Stark, John L. Markley

**Affiliations:** National Magnetic Resonance Facility at Madison, and Biochemistry Department, University of Wisconsin-Madison, Madison, WI 53706 USA

**Keywords:** NOE assignment, 3D structure determination, Client server, Semi-automation, Graphical interface for data visualization and refinement, Structure refinement and validation

## Abstract

Peak-picking Of Noe Data Enabled by Restriction Of Shift Assignments-Client Server (PONDEROSA-C/S) builds on the original PONDEROSA software (Lee et al. in Bioinformatics 27:1727–1728. doi:10.1093/bioinformatics/btr200, 2011) and includes improved features for structure calculation and refinement. PONDEROSA-C/S consists of three programs: *Ponderosa Server*, *Ponderosa Client*, and *Ponderosa Analyzer*. PONDEROSA-C/S takes as input the protein sequence, a list of assigned chemical shifts, and nuclear Overhauser data sets (^13^C- and/or ^15^N-NOESY). The output is a set of assigned NOEs and 3D structural models for the protein. *Ponderosa Analyzer* supports the visualization, validation, and refinement of the results from *Ponderosa Server*. These tools enable semi-automated NMR-based structure determination of proteins in a rapid and robust fashion. We present examples showing the use of PONDEROSA-C/S in solving structures of four proteins: two that enable comparison with the original PONDEROSA package, and two from the Critical Assessment of automated Structure Determination by NMR (Rosato et al. in Nat Methods 6:625–626. doi:10.1038/nmeth0909-625, 2009) competition. The software package can be downloaded freely in binary format from http://pine.nmrfam.wisc.edu/download_packages.html. Registered users of the National Magnetic Resonance Facility at Madison can submit jobs to the PONDEROSA-C/S server at http://ponderosa.nmrfam.wisc.edu, where instructions, tutorials, and instructions can be found. Structures are normally returned within 1–2 days.

**Electronic supplementary material:**

The online version of this article (doi:10.1007/s10858-014-9855-x) contains supplementary material, which is available to authorized users.

The growing gap between known sequences of proteins [>1.6 × 10^8^ in GenBank (Benson et al. 2008)] and 3D structures [~1 × 10^5^ in PDB (Protein Data Bank; Berman et al. 2007)] is motivating the development of improved approaches to experimental structure determination. Of the two major approaches to protein structure determination, NMR spectroscopy lags behind X-ray crystallography in terms of automated approaches. Although NMR offers the advantage of structure determination in solution with analysis of dynamic properties, fewer than one-eighth of the protein structures deposited in the PDB have been determined by NMR-spectroscopy. In the course of our participation in the CASD-NMR (Critical Assessment of automated Structure Determination by NMR; Rosato et al. 2009) and as the result of collaborations at the National Magnetic Resonance Facility at Madison (NMRFAM), we have developed a much improved version of our software package that takes as input the sequence of a protein, lists of assigned chemical shifts, and raw nuclear Overhauser effect (NOE) data sets, and returns as output a list of assigned NOE peaks and a set of three-dimensional structural models for the protein. This new software package, PONDEROSA-C/S, is based on a client–server model and offers improved performance and features (Supplementary Table S1).

PONDEROSA-C/S consists of three software programs (Fig. 1). *Ponderosa Client* enables the upload of input data via the Internet. *Ponderosa Server* accepts jobs submitted by users and distributes them to available vacant servers to balance the workload. NMRFAM currently has a cluster of six servers, but we plan to expand the services by utilizing HTCondor (High-Throughput Condor; Thain et al. 2005) to enable nearly unlimited calculation resources. *Ponderosa Server* determines distance and angle constraints, calculates 3D structures, and estimates the quality of the structures. The results are sent back to the user by e-mail within 1–2 days. *Ponderosa Analyzer* then enables users to visualize the calculated structures along with violations of input constraints. NMRFAM SPARKY distribution (Lee et al. 2009) is used to examine and refine restraints derived from NOE spectra, and PyMOL (Schrödinger et al., http://www.pymol.org) software is used to display constraints in Cartesian space. Once a set of refined constraints is determined through the use of *Ponderosa Analyzer*, they can be passed to *Ponderosa Client* for another round of structure determination. This sequential process provides a robust platform of NMR solution structure determination.

**Fig. 1:**
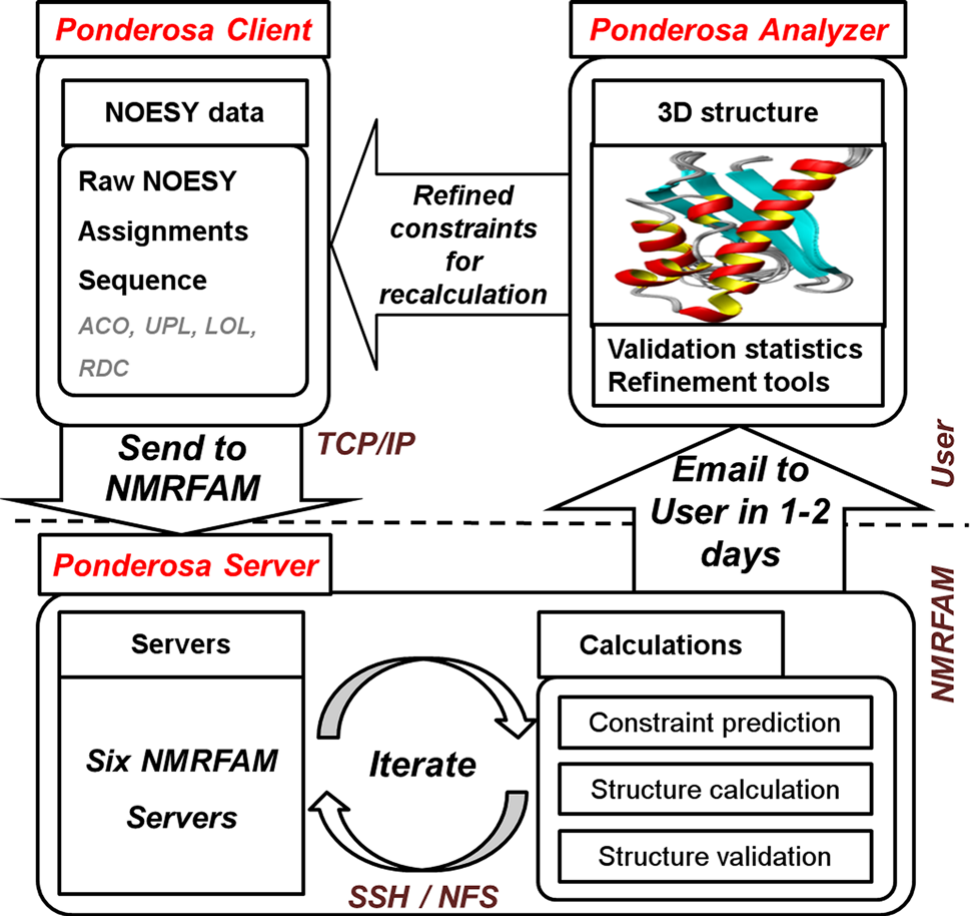
Schematic diagram of PONDEROSA-C/S illustrates how components are related and articulated

The original PONDEROSA package utilized only raw NOESY spectra in the SPARKY.ucsf file format (Goddard and Kneller 2008). In PONDEROSA-C/S, input data types have been expanded to include NOE data in NMRPIPE (Delaglio et al. 1995) format, and unrefined peak lists in XEASY (Bartels et al. 1995) or SPARKY formats (Supplementary Table S1 and Supplementary Fig. S1a). The new package can accept aromatic NOESY as well as folded NOE spectra. Residual dipolar couplings (RDCs) can be specified as well as known disulfide pairings. PONDEROSA-C/S offers three options for structure calculation: *CYANA automation* uses plain CYANA as a tool for NOE assignment and structure calculation (Güntert 2004); *PONDEROSA refinement* optimize structural quality on the basis of automatically refined lists of CYANA constraints; and *constraints only* uses the constraints specified by the user, for example, angle constraints (ACO), upper limit constraints (UPL), and lower limit constraints (LOL). If *CYANA automation* or *PONDEROSA refinement* is specified, upon receiving an input file from the user-side (Supplementary Fig. S1b), *Ponderosa Server* starts generating distance constraints from CYANA and angle constraints from TALOS-N (Shen and Bax 2013) or its relatives (Cornilescu et al. 1999; Shen et al. 2009). NOE peaks are refined as in the original PONDEROSA (Lee et al. 2011). *Ponderosa Server* can distribute the load by assigning calculations to vacant servers (Supplementary Table S1). In addition, an automatic final water refinement can be set by a server administrator. *Ponderosa Server* generates water bath and smooth torsion angle potential refinement scripts (Bermejo et al. 2012) and executes them via XPLOR-NIH (Schwieters et al. 2003) to generate energetically favorable structures. Alternatively, water bath refinement, as inspired by the RECOORD and ARIA projects (Nederveen et al. 2005; Linge et al. 2003) can be generated and executed by use of CNS (Brünger et al. 1998). All of the software packages that are part of PONDEROSA-C/S are stand-alone and can be downloaded to run on a local computer, should the user prefer not to use the server at NMRFAM.

*Ponderosa Analyzer* offers a variety of tools to validate the structural models generated. CYANA target function and violations are provided along with RDC Q factors (if RDCs were used as input). MolProbity (Chen et al. 2010) and PROCHECK (Laskowski et al. 1996) are also available for structure validation. Constraint lists and validations can be visualized with PyMOL in terms of local structure and with NMRFAM SPARKY distribution with regard to the underlying NOE spectra. The software enables constraint refinement and subsequent export to *Ponderosa Client* for structure refinement.

To evaluate the performance of PONDEROSA-C/S, we used NMR data from four proteins with structures deposited in the PDB determined by less automated methods (Supplementary Table S2). The proteins varied between 76 and 160 amino acid residues. The default PONDEROSA-C/S settings were used without manual intervention. Structure determinations took between a few hours to almost 2 days. Structures determined with PONDEROSA-C/S were compared with those determined with the original PONDEROSA software package and with structures deposited in the PDB (Supplementary Fig. S2). The statistics for the PONDEROSA-C/S structures (Supplementary Fig. S3) show that the structures determined automatically with PONDEROSA-C/S are of higher quality than those obtained with the original PONDEROSA package. In addition, the quality of the PONDEROSA-C/S structures were nearly equivalent to those determined by more manual methods and deposited in the PDB. *Ponderosa Analyzer* provides tools for the validation and further refinement of the structures. PONDEROSA-C/S currently is being used in collaborative investigations with proteins as large as 168 residues. These studies will be published separately.

## Electronic supplementary material

Below is the link to the electronic supplementary material.

Supplementary material 1 (DOCX 1934 kb)
