# Supplementary material for: PONDEROSA-C/S: client–server based software package for automated protein 3D structure determination
Source: J Biomol NMR. 2014 Sep 5;60(2):73–5. doi: 10.1007/s10858-014-9855-x (PMC4207954; doi:10.1007/s10858-014-9855-x)
Supplement: Supplementary file 1 — Supplementary material 1 (DOCX 1934 kb) [file 10858_2014_9855_MOESM1_ESM.docx]

Supplementary INFORMATION

**PONDEROSA-C/S: Client-server based software package for automated protein 3D structure determination**

Woonghee Lee^*^, Jaime L. Stark, and John L. Markley^*^

National Magnetic Resonance Facility at Madison, and Biochemistry Department, University of Wisconsin-Madison, Madison, WI 53706, USA

* To whom correspondence should be addressed

E-mail: [whlee@nmrfam.wisc.edu](mailto:whlee@nmrfam.wisc.edu), Telephone: +1-263-9498

[markley@nmrfam.wisc.edu](mailto:markley@nmrfam.wisc.edu), Telephone: +1-263-9349, Fax: +1-262-3759

**(a) (b)**

**
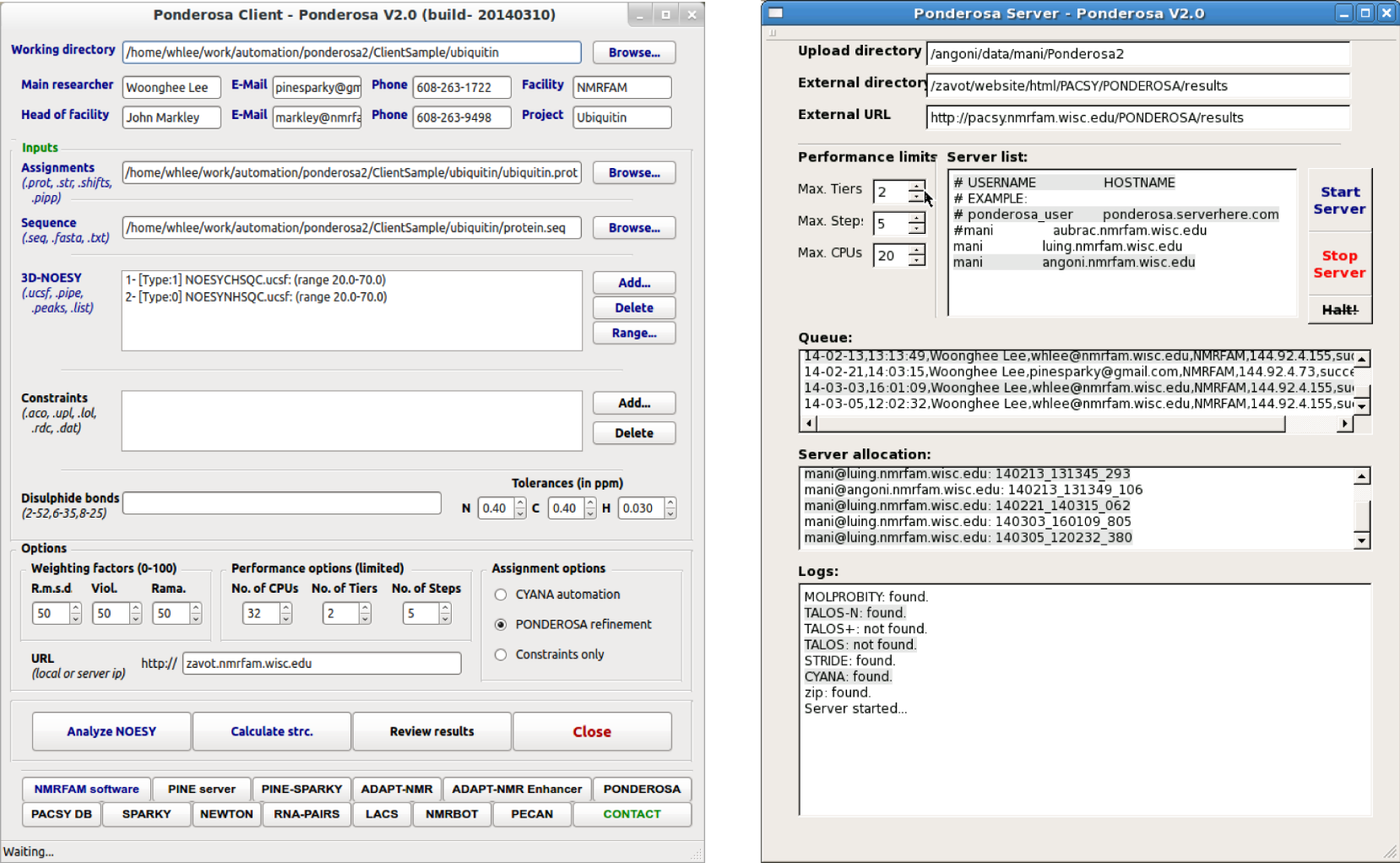
**

**{c}**


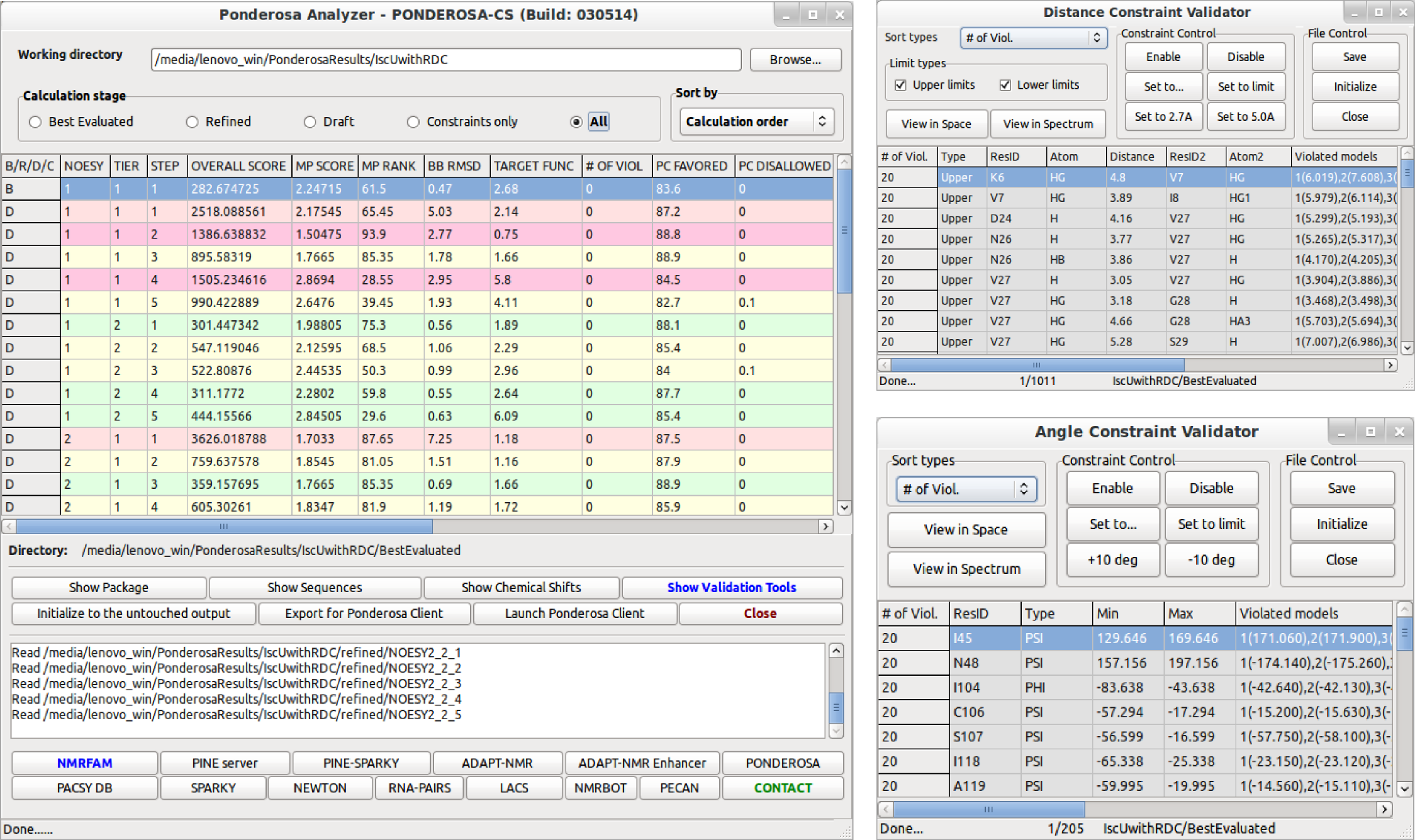


**Supplementary Fig. S1.** Screen shots of the graphical interfaces for the three programs that constitute PONDEROSA-C/S: (a) *Ponderosa Client*, (b) *Ponderosa Server*, (c) and *Ponderosa Analyzer*.

**
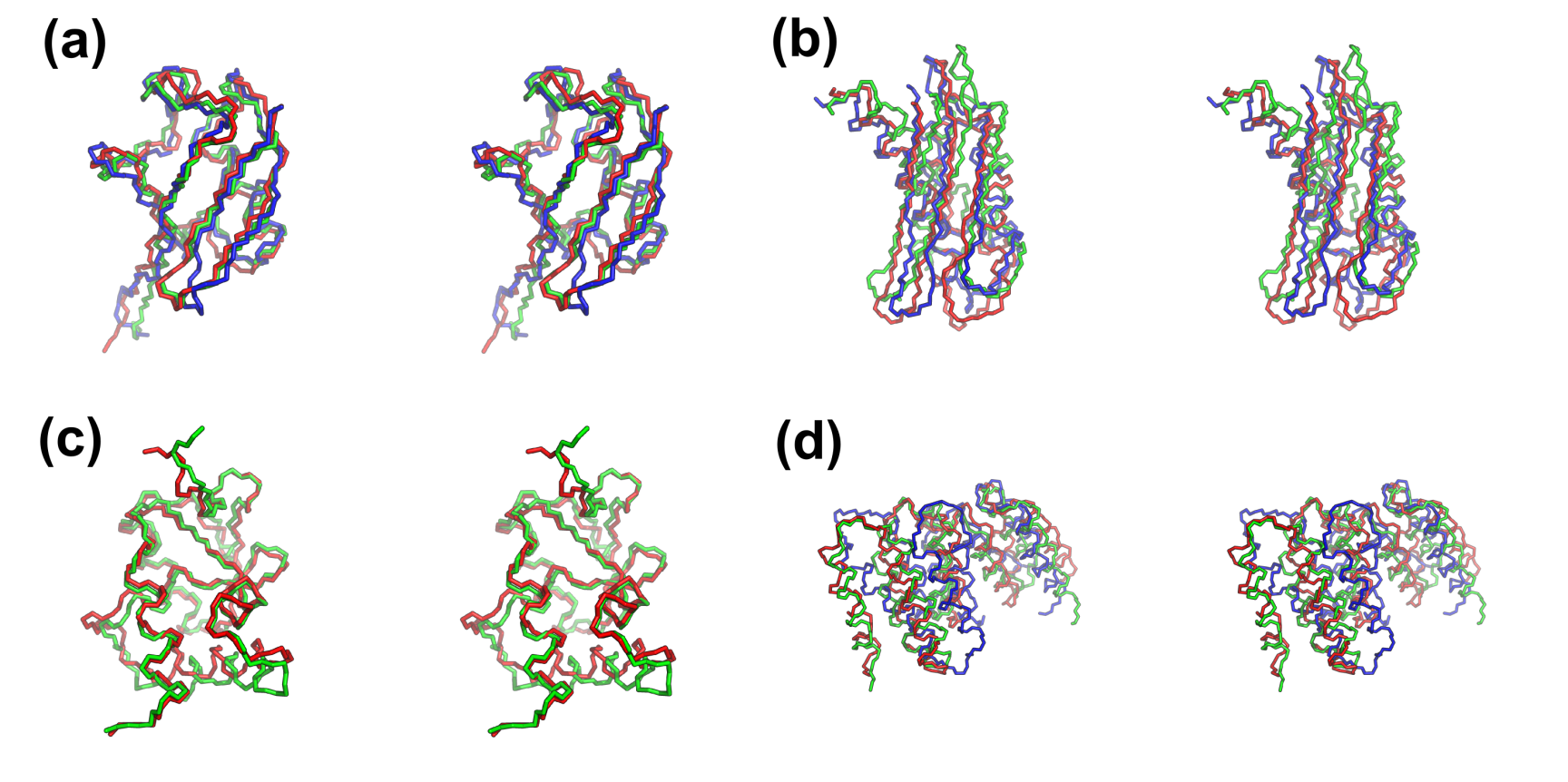
**

**Supplementary Fig. S2.** Stereo illustrations of protein structures determined by PONDEROSA-C/S without manual intervention (*green lines*). These structures are compared, where available, to those determined by the original PONDEROSA package (Lee *et al*., 2009; *blue lines*); otherwise they are compared to structures determined by less automated methods and deposited in the protein data bank (PDB) (Bernstein *et al.*, 1997; *red lines*) (Table S2). (a) human ubiquitin (Cornilescu *et al*., 1998), (b) *apo*-IscU (Kim *et al*., 2009), (c) VpR247 (Aramini *et al*., 2010), (d) HR5460A (Liu *et al*., 2011). PyMOL was used to prepare and superimpose representative structural models

**Supplementary Table S1.** Comparison of the PONDEROSA and PONDEROSA-C/S software packages.

|  | PONDEROSA | PONDEROSA-C/S |
| --- | --- | --- |
| OS | Linux | Linux, Windows, MacOSX for users Linux for servers |
| Installation^a^ | Harder (requires prerequisites) | Easier |
| Supported formats^b^ | BMRB (.str), CYANA (.aco, .upl, .lol), SPARKY (.shifts, .ucsf), XEASY (.prot, .seq) | BMRB (.str), CYANA (.aco, .upl, .lol, .rdc), FASTA (.fasta), GNOM (.dat), NMRPIPE (.pipe), PINE (.txt), PIPP (.pipp), SPARKY (.shifts, .list, .ucsf), XEASY (.prot, .seq, .peaks) |
| Nomenclature for protons | HX | HX, MX, QX |
| Supported NOESY | ^13^C-edited aliphatic NOESY ^15^N-edited NOESY | ^13^C-edited aliphatic NOESY ^13^C-edited aromatic NOESY ^15^N-edited NOESY |
| Multiple NOESY spectra of the same kind^d^ | Intensities summed (limited) | Combinatorial intensities (unlimited) |
| Analysis order of NOESY spectra | ^13^C first, ^15^N later (one-by-one) | More informative NOESY analyzed first followed by interactive hopping among spectra (well balanced) |
| Folded spectra | Not supported | Supported (unfolded automatically) |
| RDC, SAXS | Not supported | Supported (RDC) Upwardly compatible (SAXS) |
| Scoring system | Procheck based (Laskowski *et al*., 1996) | MolProbity based (Chen *et al*., 2009) |
| Validation | PonderosaUI as a viewer | Ponderosa Analyzer as a viewer, validator, and editor with support for SPARKY (NMRFAM distribution) and PyMOL |
| Refinement^e^ | Limited and manual | Fully supported (CNS, XPLOR-NIH) |
| Performance | Good | Better |

^a^PONDEROSA and stand-alone versions of PONDEROSA-C/S require installation of STRIDE (Frishman *et al*., 1995), TALOS+ (Shen *et al*., 2009), and CYANA (Güntert, 2004). These are pre-packaged for users of the NMRFAM version of PONDEROSA-C/S.

^b^Literature references for these formats: BMRB (Ulrich *et al*., 2008), CYANA (Güntert, 2004), FASTA (Pearson *et al*., 1998), GNOM (Svergun, 1992), NMRPIPE (Delaglio *et al*., 1995), PINE (Bahrami *et al*., 2009), PIPP (Garrett *et al*., 2011), SPARKY (Goddard *et al*., 2008); XEASY (Bartels *et al*., 1995).

^d^If more than one of the same kind of NOESY spectrum is uploaded, PONDEROSA combines all the intensities to generate single values whereas PONDEROSA-C/S treats each spectrum separately.

^e^Depending on which program is available, PONDEROSA-C/S automatically runs CNS or XPLOR-NIH refinement (water solvation and torsion angle database driven) (Bermejo *et al*., 2012).**Supplementary Table S2.** Four protein targets used in testing PONDEROSA-C/S test.

| Protein name | PDB code | Number of amino acid residues | Residue number of the first amino acid | Stretches of ordered residues^a^ | Average pairwise backbone RMSD^b^ to reference structure determined by conventional methods and deposited in the PDB | |
| --- | --- | --- | --- | --- | --- | --- |
|  |  |  |  |  | PONDEROSA | PONDEROSA-C/S |
| Ubiquitin (human) | 1D3Z | 76 | 1 | 2−71 | 1.24 Å (267/280)^c^ | 0.99 Å (275/280)^c^ |
| IscU(D39A) | 2KQK | 128 | 1 | 27−126 | 0.92 Å (332/408)^c^ | 0.99 Å (349/408)^c^ |
| VpR247^d^ | 2KIF | 102 | 1 | 1−101 | - | 0.92 Å (320/404)^c^ |
| HR5460A^e^ | 2LAH | 160 | 1 | 12−160 | 6.94 Å (501/596)^c^ | 3.00 Å (554/596)^c^ |

^a^Ordered residues were specified by the PSVS validation suite (Bhattacharya *et al*., 2007).

^b^PyMOL was used to determine average pairwise backbone (N, C′, C^α^, O) RMSD (root-mean-square-deviation) values for the first structural model in the family of structures. To exclude disordered regions, pairwise RMSD values greater than 2 Å were excluded.

^c^Ratio of the number of accepted atoms (those below the 2 Å cutoff) to the total number of atoms. PONDEROSA-C/S outperformed PONDEROSA by this metric in all cases.

^d^Target VpR247 was from 1^st^ round of CASD-NMR in which only refined peak lists rather than raw spectra were provided (Rosato *et al*., 2012). These refined peak lists were used as input to PONDEROSA-C/S; PONDEROSA does not accept peak lists as input.

^e^Target HR5460A from 2^nd^ round of CASD-NMR was acknowledged in the 2013 CASD-NMR meeting at Piscataway, New Jersey, as the most difficult target. PONDEROSA failed to achieve a good result with this target, but PONDEROSA-C/S performed somewhat better.

**Supplementary Table S3.** Statistics for the NMR structures of targets determined by PONDEROSA-C/S

(**A**) Human ubiquitin

| Conformationally restricting distance constraints |  |
| --- | --- |
| Short Range [(i– j) <= 1] | 874 |
| Medium Range [1 < (i – j) ≤ 5] | 275 |
| Long Range [(i – j) > 5] | 486 |
| Total | 1635 |
| Dihedral angle constraints |  |
| ϕ | 72 |
| ψ  Hydrogen-bond constraints | 75  32 |
| CYANA target function [Å]  Average rmsd to the mean CYANA coordinates [Å] | 1.34 |
| Backbone heavy atoms N, Cα, C′ (1–73) | 0.3 |
| All heavy atoms (1–73) | 0.6 |
| PROCHECK Z-scores (φ and Ψ/all dihedral angles ) | 0.12/-0.53 |
| MOLPROBITY Mean score/clash score | 2.45/23.86 |
| Ramachandran plot summary ordered residue ranges [%] |  |
| Most favored regions | 99.9 |
| Allowed regions | 0.1 |
| Disallowed regions | 0 |
| Average number of distance constraint violations per CYANA conformer |  |
| 0.2 – 0.5 Å | 4 |
| > 0.5 Å | 0 |
| Average number of angle constraint violations per CYANA conformer |  |
| > 10° | 0 |

(**B**) IscU(D39A)

| Conformationally restricting distance constraints |  |
| --- | --- |
| Short Range [(i– j) <= 1] | 659 |
| Medium Range [1 < (i – j) ≤ 5] | 240 |
| Long Range [(i – j) > 5] | 308 |
| Total | 1207 |
| Dihedral angle constraints |  |
| ϕ | 101 |
| ψ  Hydrogen-bond constraints | 104  44 |
| CYANA target function [Å]  Average rmsd to the mean CYANA coordinates [Å] | 0.21 |
| Backbone heavy atoms N, Cα, C′ (19–61, 68-125) | 0.9 |
| All heavy atoms (19–61, 68-125) | 1.3 |
| PROCHECK Z-scores (φ and Ψ/all dihedral angles ) | 1.26/0.71 |
| MOLPROBITY Mean score/clash score | 1.77/10.31 |
| Ramachandran plot summary ordered residue ranges [%] |  |
| Most favored regions | 99.8 |
| Allowed regions | 0.2 |
| Disallowed regions | 0 |
| Average number of distance constraint violations per CYANA conformer |  |
| 0.2 – 0.5 Å | 1 |
| > 0.5 Å | 0 |
| Average number of angle constraint violations per CYANA conformer |  |
| > 10° | 0 |

(**C**) VpR247

| Conformationally restricting distance constraints |  |
| --- | --- |
| Short Range [(i– j) <= 1] | 941 |
| Medium Range [1 < (i – j) ≤ 5] | 275 |
| Long Range [(i – j) > 5] | 379 |
| Total | 1595 |
| Dihedral angle constraints |  |
| ϕ | 86 |
| ψ  Hydrogen-bond constraints | 90  38 |
| CYANA target function [Å]  Average rmsd to the mean CYANA coordinates [Å] | 0.51 |
| Backbone heavy atoms N, Cα, C′ (2–101) | 0.6 |
| All heavy atoms (2–101) | 1.1 |
| PROCHECK Z-scores (φ and Ψ/all dihedral angles ) | 1.22/1.24 |
| MOLPROBITY Mean score/clash score | 1.83/10.93 |
| Ramachandran plot summary ordered residue ranges [%] |  |
| Most favored regions | 98.2 |
| Allowed regions | 1.7 |
| Disallowed regions | 0.1 |
| Average number of distance constraint violations per CYANA conformer |  |
| 0.2 – 0.5 Å | 3 |
| > 0.5 Å | 0 |
| Average number of angle constraint violations per CYANA conformer |  |
| > 10° | 0 |

(**D**) HR5460A

| Conformationally restricting distance constraints |  |
| --- | --- |
| Short Range [(i– j) <= 1] | 1737 |
| Medium Range [1 < (i – j) ≤ 5] | 958 |
| Long Range [(i – j) > 5] | 709 |
| Total | 3404 |
| Dihedral angle constraints |  |
| ϕ | 136 |
| ψ  Hydrogen-bond constraints | 142  67 |
| CYANA target function [Å]  Average rmsd to the mean CYANA coordinates [Å] | 3.02 |
| Backbone heavy atoms N, Cα, C′ (12–159) | 0.5 |
| All heavy atoms (12–159) | 0.9 |
| PROCHECK Z-scores (φ and Ψ/all dihedral angles ) | 1.85/0.24 |
| MOLPROBITY Mean score/clash score | 2.90/38.74 |
| Ramachandran plot summary ordered residue ranges [%] |  |
| Most favored regions | 98.0 |
| Allowed regions | 2.0 |
| Disallowed regions | 0.0 |
| Average number of distance constraint violations per CYANA conformer |  |
| 0.2 – 0.5 Å | 9 |
| > 0.5 Å | 0 |
| Average number of angle constraint violations per CYANA conformer |  |
| > 10° | 0 |

**Supplementary Equation S1.** Scoring system used for estimating structure quality:

$$S =\sum_{k=1}^{n} R\left( k \right)W(k)$$

where *S* is the pseudo energy for the structure (lower values are better), and *k* represents each criterion used in judging structure quality with ranking (*R*) and weighting factors (*W*) set by the user (default values rank and weight the criteria equally). These criteria are: 1) goodness of fit to the Ramachandran plot in MolProbity or PROCHECK, 2) the number of constraint violations, and 3) the backbone RMSD.

**REFERENCES**

Aramini,J.M. et al. (2010) Structural Basis of O6-Alkylguanine Recognition by a Bacterial Alkyltransferase-like DNA Repair Protein. J Biol Chem, 285, 13736-13741.

Bartels,C. et al. (1995) The program XEASY for computer-supported NMR spectral analysis of biological macromolecules. J. Biomol. NMR, 6, 1-10.

Bermejo,G.A. et al. (2012) Smooth statistical torsion angle potential derived from a large conformational database via adaptive kernel density estimation improves the quality of NMR protein structures. Protein Sci., 21, 1824-1836.

Bernstein,F.C. et al. (1977) The Protein Data Bank: a computer-based archival file for macromolecular structures. J. Mol. Biol., 112, 535-542.

Bhattacharya,A. et al. (2007) Evaluating protein structures determined by structural genomics consortia. Proteins, 66, 778-795.

Chen,V.B. et al. (2010) MolProbity: all-atom structure validation for macromolecular crystallography. Acta Crystallogr. D Biol. Crystallogr., 66, 12-21.

Cornilescu,G. et al. (1998) Validation of Protein Structure from Anisotropic Carbonyl Chemical Shifts in a Dilute Liquid Crystalline Phase. Journal of the American Chemical Society, 120, 6836-6837.

Delaglio,F. et al. (1995) NMRPipe: a multidimensional spectral processing system based on UNIX pipes. J. Biomol. NMR, 6, 277-293.

Frishman,D. and Argos,P. (1995) Knowledge-based protein secondary structure assignment. Proteins, 23, 566-579.

Garrett,D.S. et al. (2011) A common sense approach to peak picking in two-, three-, and four-dimensional spectra using automatic computer analysis of contour diagrams. 1991. J. Magn. Reson., 213, 357-363.

Goddard, T.D. and Kneller, D.G. SPARKY 3. University of California, San Francisco.

Güntert,P. (2004) Automated NMR structure calculation with CYANA. Methods Mol. Biol., 278, 353-378.

Kim,J.H. et al. (2009) Structure and dynamics of the iron-sulfur cluster assembly scaffold protein IscU and its interaction with the cochaperone HscB. Biochemistry, 48, 6062-6071.

Laskowski,R.A. et al. (1996) AQUA and PROCHECK-NMR: programs for checking the quality of protein structures solved by NMR. J. Biomol. NMR, 8, 477-486.

Lee,W. et al. (2011) PONDEROSA, an automated 3D-NOESY peak picking program, enables automated protein structure determination. Bioinformatics, 27, 1727-1728.

Lee,W. et al. (2009) PINE-SPARKY: graphical interface for evaluating automated probabilistic peak assignments in protein NMR spectroscopy. Bioinformatics, 25, 2085-2087

Liu, G., et. al. (2011) Solution NMR Structure of Mitotic checkpoint serine/threonine-protein kinase BUB1 N-terminal domain from Homo sapiens, Northeast Structural Genomics Consortium Target HR5460A (Methods Development). DOI:10.2210/pdb2lah/pdb

Pearson,W.R. and Lipman,D.J. (1988) Improved tools for biological sequence comparison. Proc. Natl. Acad. Sci. U.S.A., 85, 2444-2448.

Schrödinger , The PyMOL Molecular Graphics System, Version 1.2r3pre, LLC.

Svergun,D.I. (1992) Determination of the regularization parameter in indirect-transform methods using perceptual criteria. Journal of Applied Crystallography, 25, 495-503.

Ulrich,E.L. et al. (2008) BioMagResBank. Nucleic Acids Res., 36, D402-408.
